# Rule-based induction method for haplotype comparison and identification of candidate disease loci

**DOI:** 10.1186/gm320

**Published:** 2012-03-19

**Authors:** Sirkku Karinen, Silva Saarinen, Rainer Lehtonen, Pasi Rastas, Pia Vahteristo, Lauri A Aaltonen, Sampsa Hautaniemi

**Affiliations:** 1 Research Programs Unit, Genome-Scale Biology, and Institute of Biomedicine, Biochemistry and Developmental Biology, University of Helsinki, Haartmaninkatu 8, Helsinki, FIN-00014, Finland; 2 Research Programs Unit, Genome-Scale Biology, and Haartman Institute, Department of Medical Genetics, University of Helsinki, Haartmaninkatu 8, Helsinki, FIN-00014, Finland; 3 Department of Biological Sciences, University of Helsinki, Viikinkaari 1, FIN-00014, Finland; 4 Department of Computer Science, University of Helsinki, Gustaf Hällströmin katu 2b, FIN-00014, Finland

## Abstract

There is a need for methods that are able to identify rare variants that cause low or moderate penetrance disease susceptibility. To answer this need, we introduce a rule-based haplotype comparison method, Haplous, which identifies haplotypes within multiple samples from phased genotype data and compares them within and between sample groups. We demonstrate that Haplous is able to accurately identify haplotypes that are identical by descent, exclude common haplotypes in the studied population and select rare haplotypes from the data. Our analysis of three families with multiple individuals affected by lymphoma identified several interesting haplotypes shared by distantly related patients.

## Background

One of the most important goals in biomedical research is to identify genes that predispose humans to diseases, such as cancer. To facilitate the identification of these genes, a number of genome-wide approaches have been suggested, such as genetic linkage and genome-wide association (GWA) methods [1]. The linkage methods have revealed several high penetrance disease susceptibility loci [1], whereas GWA studies have been useful in the 'common disease - common allele' model [2]. However, neither approach is well suited to tackle moderate penetrance susceptibility because such a condition rarely results in large pedigrees, with few or no phenocopies, convenient for linkage analysis. As the GWA approaches cannot detect these presumably rare alleles, there is clearly a need for methods that are able to identify loci where such variants could be located. Evolutionarily recent, and thus rare, mutations are usually conveyed in a pedigree by a shared haplotype. Therefore, detection of such haplotypes can lead to the identification of rare or moderate penetrance variants behind disease susceptibility.

We introduce here Haplous, a novel computational approach that uses phased genotype data, such as genome-wide SNPs, to identify and prioritize genomic regions likely to be inherited from a common ancestor. The central idea of our approach is to use haplotypes, instead of single alleles, and rank them based on expert-defined rules that determine the haplotypes shared in heterozygous and homozygous forms. As the identification of haplotypes has been recognized as useful for revealing disease predisposing genes, several haplotype association methods have been developed [3-10]. These methods include detection of haplotype diversity and statistical association tests. Haplotypes can be detected with fixed or variable length sliding window [7,11,12], haplotype blocks [13], haplotype clustering [9], a cladistic approach [10] or considering non-contiguous haplotypes [5,8]. Some haplotype analysis methods are feasible in genome-wide settings [9,11,13], although several are intended only for smaller datasets [6,12]. Many haplotype-based approaches essentially aim to identify identical by descent (IBD) regions between samples [14-16].

To our knowledge, Haplous is the first method that uses a rule-based approach to identify rare haplotypes shared by multiple individuals from genome-wide data. To identify a rare disease-associated haplotype with a statistical test would require thousands of samples, which in general is not feasible. The rule-based induction takes advantage of prior knowledge and research hypothesis to rank the identified regions, which allows analysis of small cohorts. The main objective of Haplous is to allow analysis and comparison of phased haplotypes; it thus goes further than simply identifying IBD regions from pairs of individuals. Haplous is freely available with a user guide [17].

We used here three synthetic case studies to demonstrate the ability of Haplous to identify rare shared haplotypes. First, we used the HapMap database [18] to assess the true positive (TP) and false positive (FP) rates when haplotype estimation has introduced uncertainty to the haplotype data. We chose individuals from the HapMap database whose haplotype phases were deterministically estimated using pedigree (phase-known) data, and compared those data to a setting in which their haplotype phases were estimated using a population based method (phase-predicted). Second, we simulated datasets from an extended pedigree with a mutation inherited from a common ancestor and a dataset for a healthy non- related population, and identified the mutation locus from the data. We also compared Haplous with an existing method [14]. Third, we studied the robustness of Haplous in settings where the parameters or sample grouping are incorrect.

In order to show that Haplous is applicable to an experimental setting where the aim is to identify predisposition loci for a complex disease using familial information, we analyzed three Finnish lymphoma families with putative genetic lymphoma predisposition, as several individuals in these families have been affected by Hodgkin lymphomas (HLs) and non-Hodgkin lymphomas (NHLs). The incidence of HL is about 3 in 100,000 per year, and most cases are sporadic. However, many familial clusters of HL have been reported, and large epidemiological studies have confirmed the increased familial risk associated with HL [19]. Recently, *KLHDC8B *and *NPAT *have emerged as candidate HL predisposition genes [20,21], but these preliminary results have not yet been confirmed. Thus, familial HL is an example of a disease in which a hereditary component is apparent but little is known about its molecular background. In our analysis, Haplous suggested several loci that may contain lymphoma-associated genes, and the genes located in the best-scoring regions were further prioritized with the SNPs3D text mining method [22].

## Materials and methods

### Haplous

Haplous is designed to detect haplotypes inherited by individuals who have the same familial disease predisposition and a distantly related common ancestor. It can also be used to compare haplotypes shared by a particular group of affected individuals to haplotypes of unaffected non-related controls. The same haplotype is found from several samples due to a common ancestor or a random event. That is, the haplotype is IBD (common ancestor) or identical by state (IBS; random event). The IBD haplotypes can be identified by filtering against the IBS haplotypes in matched controls and focusing on long haplotypes.

Haplous searches for a shared haplotype (SH) between individuals by using a sliding window and compares the SHs among sample sets, such as cases and controls. The comparison is based on rules, which formulate homozygous and heterozygous haplotype composition within and between sample groups. Each SH is assigned a score that allows ranking and prioritization. The score is based on the length of the SH and its abundance in cases and controls. The input to Haplous is phased genotypes and outcome is ranked lists of SHs and corresponding chromosomal regions.

Haplous is implemented in Java, and it is freely available as an independent Java library. In addition, it is included as a pipeline in the Anduril bioinformatics workflow engine [23]. Anduril compliance allows straightforward integration of Haplous analysis and results to other studies. The Haplous Java library, Anduril components and the user guide are freely available [17].

### Haplous parameters

Haplous uses phased haplotypes and predicts the SHs by comparing consecutive SNP alleles with each other using a fixed-sized sliding window with user-adjustable parameters for window size (*w*), mismatches (*m*), and length of identical regions (*l*). Each sample has the phased SNPs for maternal and paternal chromosomes, which are effectively two allele vectors for each sample. Each allele vector in turn is used as a reference to which all other allele vectors are compared. The window of *w *markers is slid over the reference vector and compared to an allele vector, and windows having at most *m *differences are identified as SHs. Shorter identical regions of *l *markers are identified as SHs as well.

**Figure 1:**
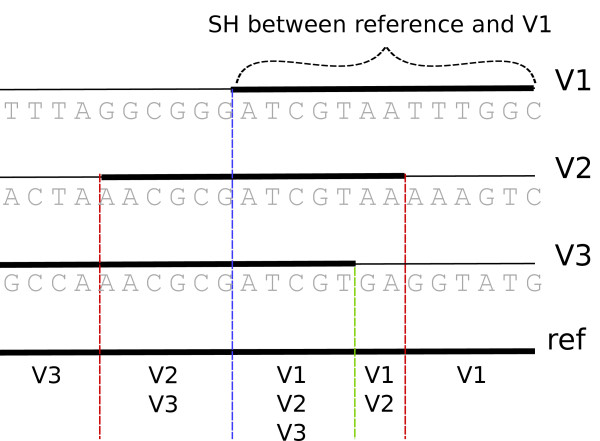
**Haplous identifies the shared haplotypes between the reference vector and all other allele vectors**. This figure shows an example of data rows (nucleotides) that are the phased maternal and paternal chromosomes of two samples, and which are treated as allele vectors. Haplous identifies shared haplotypes (SHs) between these allele vectors and collapses a list of regions and allele vectors that have a SH in that region.

For each reference vector, this produces a pair-wise map of SHs between all other allele vectors, and all the pairs are collapsed into a single data structure that identifies the vectors that have the same SH and the location of this SH. This process is illustrated in Figure 1. This structure enables an easy lookup of all samples in each SH. The SHs are always defined against the reference vector. Haplous allows mismatches in SHs, meaning that if an allele vector *A *has a SH with an allele vector *B *(*A *= *B*) and if *B *= *C*, it still might be that *A *≠ *C*. Thus, having multiple pair-wise SHs in a region does not mean that all the allele vectors are the same. Missing markers are treated as matches. The maternal and paternal vectors in each sample are also compared with each other, which reveals the location of homozygous regions.

The running time and the memory requirements of Haplous grow quadratically with the number of samples and linearly with the number of markers. Therefore, Haplous is applicable in genome-wide studies in rare diseases, where the number of samples is less than 500. For larger sample sizes, Haplous allows the user to define those samples that are used as a reference in the comparison, and the rest of the samples are skipped. This is a useful option in the case-control settings, since Haplous can be used to compare cases to controls but skip the control-case and the control-control comparisons. Additionally, each sample can be analyzed independently from each other, which enables parallelization of each run. Haplous is also implemented on an Anduril [23] bioinformatics workflow engine that automatically parallelizes the execution. Thus far, Haplous has been used to analyze datasets of 930 cases and 960 controls (data not shown).

Haplous gives an estimate for informativeness of each SH. This informativeness describes how rare a given SH is. Informativeness is defined as a joint probability of the alleles in SH estimated from the allele frequencies by multiplying allele frequencies *a_i _*from the first marker of SH (*i*) to the last marker (*n*), that is, $\prod_{i}^{n}P\left(a_{i}\right)$. The user may set a threshold (*t*) for informativeness. In this case, only those SHs that have informativeness below *t *are included in the results. The value of informativeness is between zero and one. Zero denotes that the alleles of SH could never be seen in the population, while one means that alleles of SH are always observed in that region and thus the region is completely uninformative.

Simply scanning all SHs that meet the criteria would produce a huge list of SHs that are abundant in the population but not particularly interesting regarding the phenotype in question. To find the interesting SHs, we make use of the expert knowledge of the user: the user defines the rules that determine which SHs are interesting. The rules were used to define the features that an interesting SH needs to have, and these features are defined for cases and controls separately. If a SH has these features, it is considered interesting. The rules are set as thresholds for the number of cases or controls that share the SH.

These rules can be better understood through an analogy with basic parametric linkage analysis. The main differences are that in Haplous the 'parameters' are presented as counts instead of percentages, and IBD sharing expectations between families can be controlled at the same time. We need to find the IBD haplotype carrying a predisposing mutation that segregates with the disease trait. These rules include the inheritance model (dominant and recessive model - that is, heterozygotes and homozygotes), assumed penetrance (proportion of mutation-carrying individuals who have the disease in question - that is, the number of cases and controls sharing the same haplotype), phenocopies (the number of cases that do not share the same haplotype) and mutation frequency (the total number of cases and controls that share the same haplotype). Using the rules, the user can tune the parameters to correspond to the hypothesis of the current analysis.

**Figure 2:**
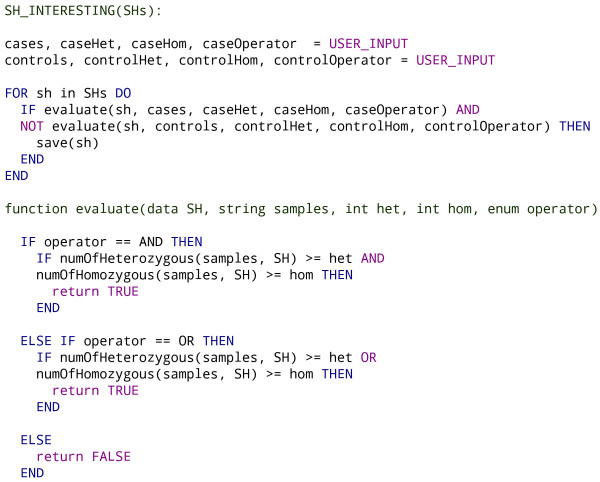
**Pseudo-code for interesting shared haplotype inference**. The interesting shared haplotypes (SHs) are inferred based on the user input, which defines the names for cases and controls as well as the thresholds for the number of homozygous and heterozygous samples in the interesting SH. Each SH is evaluated using the *evaluate *function, which takes the user parameters as an input. The evaluation is done for case and control thresholds separately, but the Boolean result value is negated for controls. The *evaluate *function calculates how many times the named samples (cases or controls) share the SH, and if the thresholds are exceeded, it returns a true value, otherwise it returns a false value.

The pseudo-code of this inference is given in Figure 2. Briefly, the inference algorithm takes the thresholds and list of cases and controls as an input, calculates the number of cases and controls sharing each SH, and evaluates whether a SH has the features of an interesting SH taking into account both the cases and controls. These evaluations for cases and controls are produced with the same function but the return value is negated for the control rule. The rules follow a natural deduction, an example of which could be: 'the SH is interesting if it is shared by one or more homozygous case samples and not shared by any control samples'.

After filtering the most interesting SHs, the result set may still include many regions that are almost equally promising for further studies. Haplous gives scores for SHs, which are stored in a file with information about the range, score and samples sharing the particular haplotype. This allows straightforward identification and post-processing of the most interesting homozygous and heterozygous chromosomal regions.

The score calculated by Haplous emphasizes the number of cases and controls that share the haplotype as well as the length of the SH. The score is calculated according to the formula *M*(*C_a _*- *C_o_*), where *M *is the number of markers in the chromosomal region of a SH, *C_a _*is the number of times cases share the SH and *C_o _*is the number of times controls share the SH. Note that in the case of homozygous loci, each homozygous sample shares the SH twice, which increases either *C_a _*or *C_o_*. Instead of a specific haplotype, we assign a score for the chromosomal region carrying the different haplotypes. This score is assigned similarly *M_r_*(*C_ar _*- *C_or_*), where *C_ar _*is the number of cases and *C_or _*the number of controls having any SH in a given marker. *M_r _*is the number of markers in the chromosomal region that receives the same score from (*C_ar _*- *C_or_*). The upper or lower limits of the scores depend on the parameter values.

### HapMap data

We used the HapMap phase 3 European population (CEU) chromosome 12 [18] phase-known dataset that has been created for each family trio in the database. Computational methods are needed to estimate haplotype phases from high-throughput SNP data. In many cases, genotypes of the family members are not available but instead population data are used as a reference [24]. However, estimates based on family genotypes are more accurate since inheritance of most of the SNPs can be estimated based on the pedigree. The HapMap database provides unphased genotypes and corresponding haplotypes inferred from the families [18]. In HapMap, the phases of 94% of SNPs are known through the family information in the parents-child trios [25]. On average, 28% of the SNPs are heterozygous, and the trios reveal the phase for 80% of the heterozygous SNPs. We treat the more accurate family-based haplotypes as phase-known haplotypes, and use Haplous to compare them with the estimated phase-predicted haplotypes of the same HapMap samples, using Haplous.

Here we used only data from parents, as children convey redundant information. To create a population of unrelated individuals for the haplotype phase estimation, we randomly picked one individual from each family and used their unphased genotypes. Next, we selected SNPs that were present in the phase- known and unphased datasets. These formed a dataset of 60,704 SNPs for a population of 41 unrelated individuals. The phase-predicted haplotypes were estimated with the HaploRec software [24] based on the 41 population samples. The 29 samples present in the phase-known and the phase-predicted datasets were selected to test Haplous performance, and from these samples we created two datasets, one having phase-known and the other phase-predicted haplotypes.

### Data simulation

We used the simulation software GENOME [26] to generate 6,000 SNP haplotypes from a single chromosome spanning 78 cM. The population had an effective population size of 100,000 with a mutation rate of 10^-8 ^per generation. From this population, we chose 4,068 SNPs that matched best to the Illumina SNP map of human chromosome 22 and had a minimum allele frequency of 0.05 or more. Randomly chosen 1,500 haplotypes were used as the founder haplotypes for the affected pedigree, and 500 other randomly chosen haplotypes were paired as 250 healthy controls.

We created the pedigree by selecting random members *A *and *B *from both lymphoma families 2 and 3. Both *A *and *B *had at most one direct ancestor, and their alleles could be inherited by at least 20% of the youngest generations. The new family members in this pedigree were added so that *A *and *B *had a common ancestor 10 to 27 generations away from the youngest members.

The mutated allele was inserted into the common ancestor and the rest of the founders were non-carriers. For both families, we chose ten random paths from the youngest individuals to the common ancestor and forced the mutation to be passed through generations in these paths. Then we used our own simulator to simulate the inheritance of non-founder alleles from the oldest generation to the youngest based on the genetic map of chromosome 22.

The individuals from the youngest generation who had the mutation were inserted into the case dataset. The mutation allele was set to the same allele in all the final samples. This simulation was repeated 100 times, each time varying the position of the mutation.

### Lymphoma data

**Figure 3:**
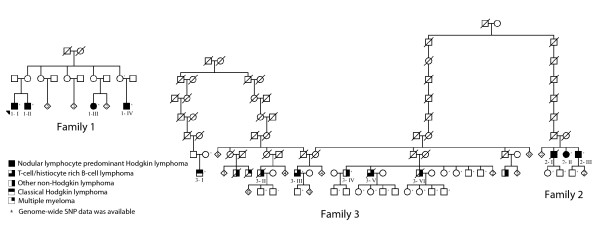
**The Finnish lymphoma families in the lymphoma study**. Family 1: the previously reported Finnish family where four cousins have had the rare subtype of Hodgin lymphoma (HL), nodular lymphocyte predominant Hodgkin lymphoma (NLPHL), in their early twenties. Family 2 and family 3: the lymphoma families from northern Finland that have a common ancestor. The patients and unaffected family members from whom genome-wide SNP data were available for haplotype determination and Haplous analysis are depicted. Numbers within diamonds indicate numbers of children. Circles, females; squares, males; slashes through symbols, deceased. Pedigrees have been slightly modified for confidentiality.

Blood-derived DNA was collected from nine lymphoma patients, of whom six had nodular lymphocyte predominant Hodgkin lymphoma (NLPHL) and three had either T-cell/histiocyte rich B-cell lymphoma (TCRBCL), NHL or classical Hodgin lymphoma (cHL). When possible, samples were also collected from the children or parents of these patients for phase determination (Figure 3). Samples were also collected from the children and siblings of four deceased lymphoma patients, one of whom had had NLPHL, one TCRBCL and two NHL (Figure 3). Altogether, 29 samples were available, of which 20 were from unaffected family members. The slightly modified pedigrees and sample information are shown in Figure 3. The samples and patient information were obtained with approval from the Ethics committees of the Helsinki University Central Hospital and Hospital District of Helsinki and Uusimaa (Dnro 408/13/03/03/2009). All blood samples were derived after a signed informed consent in accordance with the Declaration of Helsinki. Genotypes from 250 unaffected Finnish control individuals were also available from the Nordic Center of Excellence in Disease Genetics control database [27].

### Lymphoma data processing

Genome-wide SNP data were available from four lymphoma patients in family 1, two in family 2, and three in family 3, as well as from 20 unaffected family members (Figure 3). In the cases of four lymphoma patients whose DNA was not available, SNP data from their children and siblings were used to define their haplotypes. This created uninformative gaps in the haplotype data of the deceased individuals. In this study, however, we decided to consider all the uninformative regions as SHs in the haplotype screening in order not to lose any information.

Genomic DNA extracted from 29 blood samples was used for SNP genotyping with the Illumina's HumanCNV370 -Duo DNA Analysis BeadChip using Infinium Assay (Illumina Inc., San Diego, CA, USA). Genotyping was performed according to the manufacturer's standard protocol in the Institute for Molecular Medicine Finland (FIMM) Genome and Technology Centre (Finland). Genotype calling was carried out with BeadStudio software (Illumina Inc.) using the default GenCall score cutoff of 0.15. All samples passed the quality filtering. SNP genotypes were exported from BeadStudio to the Progeny database (Progeny Software LLC, South Bend, IN, USA), in which pedigree, phenotype, sample and SNP data were integrated. Mendelian error checking was performed on the genotype data using tools integrated in Progeny. The markers with Mendelian errors were removed from further analysis. Pedigree-based haplotypes were constructed using Merlin [28] with the '--best' mode, which estimates the most likely haplotype vector. For the haplotype estimation, the large pedigree of family 3 was first split into smaller overlapping sub-pedigrees. Unlikely genotypes that cause double recombinants were predicted with the Merlin error detection tool and subsequently excluded from the final analysis. The haplotypes for controls were estimated using HaploRec [24] with default parameters and the chromosome split into regions 500 markers long that overlapped by 10 markers and had an extra 20 markers at the tails of each split. In the phase- known family-based haplotypes the uninformative loci were transformed into missing markers.

### Analysis of simulated data

From each 100 simulated datasets, we selected the first 11 cases for the analysis and used all controls from each simulated dataset. The Haplous SH scan was executed using parameters '*m *= 0, *w *= {20,30,50,100,150,180,200}'. The rules for controls were (USER INPUT = {controlHet = 4, controlHom = 1, controlOperator = OR}) and for cases it was varied from (USER INPUT = {caseHet = {1,2,3, caseHom = {1,2,3}, caseOperator = AND}) to (USER INPUT = {caseHet = {1,2,3,4,5,6,7,8,9,10,11}, caseHom = {1,2,3,4,5,6,7,8,9,10,11}, caseOperator = AND}). The rules (USER INPUT = {caseHet = {1,2,3,4,5,6}, caseHom = {1,2,3,4,5,6}, caseOperator = AND}) are comparable with the lymphoma analysis, and they were used for the analysis of Haplous robustness (Tables 1 and 2). BEAGLE was executed using the parameter 'fastibd = true'.

**Table 1:** Summary of the results from the simulated dataset using different numbers of controls

	Number of controls				
	
	10	60	110	160	210
Percentage of results that included the mutation (%)	100	100	100	100	100
Percentage of mutation loci in the top hit (%)	46	43	37	38	37
Mean length of haplotypes (number of SNPs)	629	546	441	410	383

The number of controls was varied from 10 to 210, and the number of cases was kept at 11. We analyzed the data using a 100 SNP window and a threshold of six for cases and three for controls. The precision suffered with the decrease in the number of controls. The percentages of datasets that included the mutation, the percentage of mutated haplotypes as the top hit and mean length of haplotypes were calculated from the 100 simulated datasets.

**Table 2:** Summary of the results from the simulated dataset mixing cases and controls

	Number of cases/number of controls in case dataset									
	
	1/10	2/9	3/8	4/7	5/6	6/5	7/4	8/3	9/2	10/1
Percentage of datasets that had haplotypes (%)	0	0	0	0	12	97	99	99	99	100
Percentage of results that included the mutation (%)	0	0	0	0	8	99	98	98	100	99
Percentage of mutation loci in the top hit (%)	0	0	0	0	100	67	53	43	35	32
Mean length of haplotypes (number of SNPs)	0	0	0	0	163	443	428	397	377	355

The controls and cases were mixed into the same case-dataset at different ratios, and then analyzed using a 100 marker window and requiring six cases and three controls to share the shared haplotype (SH). When the number of controls was seven or more, no haplotypes were found. If the dataset had more cases, mutated haplotypes were identified with high frequency. For the borderline case of five cases and six controls, Haplous discovered SHs from 12% of datasets, and one of these had the mutation, which was, however, the top hit in that dataset. The percentages of datasets that included any haplotypes or the mutated haplotypes and the percentage of mutated haplotypes as the top hit were calculated from the 100 simulated datasets. The mean length of haplotypes in number of SNPs was also calculated from the 100 simulated datasets.

### Lymphoma analysis by Haplous

**Figure 4:**
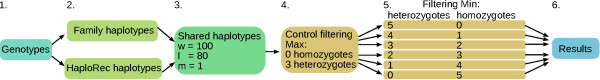
**Shared haplotype extraction pipeline**. The shared haplotype (SH) extraction workflow comprised six stages. Stage 1: the genotype calls were produced using the Illumina SNP bead arrays and transformed into data matrices. Stage 2: the haplotypes were estimated for the cases with Merlin by using the family information, and for the population controls by using the HaploRec software. Stage 3: all SHs were extracted from cases and controls. Stage 4: SHs were filtered to exclude SHs shared by at least four heterozygous or one homozygous control. Stage 5: pre-filtered SHs were then filtered further in six parallel analyses with thresholds that discover SHs present in at least five lymphoma cases. Stage 6: the ten highest scoring hits more than 30 markers long were combined from each filtering in stage 5.

A schematic of the six-staged lymphoma data analysis is presented in Figure 4. In stage 1, genotypes were imported to the analysis. In stage 2, the haplotypes were estimated from the genotypes. In stage 3, haplotypes of the cases and controls were combined and all SHs were extracted from the data using the following parameters: window size 100, no more than one mismatch within a window and identical haplotype length 80 (*w *= 100, *m *= 1 and *l *= 80). Informativeness was not considered (*t *= 1). In stage 4, SHs present in the controls were excluded by filtering, allowing the maximum of three SHs as heterozygous or none in homozygous form (USER INPUT = {controlHet = 4, controlHom = 1, controlOperator = OR}). At this stage we did not set any threshold for lymphoma cases. In stage 5, we applied in parallel six different rules to identify five SHs in cases in all conformations. As the identified families do not provide a clear clue to the mode of inheritance, we varied the number of required heterozygous SHs (hetSHs) and homozygous SHs (homSHs) in cases (USER INPUT = {caseHet = {0,1,2,3,4,5}, caseHom = {0,1,2,3,4,5}, caseOperator = AND}). In stage 6, we selected the ten highest scoring SHs that are more than 30 markers long.

The filtering breaks the SHs into shorter segments based on samples that share the SHs. Therefore, SHs encompassing at least 30 SNPs were considered to be sufficiently long to be genetically interesting, that is, to represent potential IBD haplotypes. The SHs were filtered six times, and the ten highest scoring hits from each run were examined in more detail. Region boundaries were retrieved using SNP identifiers and a list of genes located in these regions was collected from the Ensembl database (release 59) [29]. A downstream analysis of these genes was performed. Genes that had a UniProt identifier were considered as protein coding. In order to find candidate genes that could be interesting considering what is known about their function in the literature, we performed a SNPs3D [22] search for lymphoma-related features. The search terms were 'nodular lymphocyte predominant Hodgkin lymphoma', 'T-cell rich B-cell lymphoma', 'histiocyte rich B-cell lymphoma', 'non-Hodgkin lymphoma', 'Hodgkin lymphoma' and 'B-cell'. We also used three known NHL or NLPHL related genes (*BCL6 *[30,31], *A20 *[32] and *SOCS1 *[33]) as search words as well as 'NFkB', a pathway that appears to be activated in both NLPHL and non-Hodgkin lymphomas [31,33,34].

## Results

### True positive and false positive rates of Haplous using HapMap data

The haplotype phase is not seen directly from the SNP data, and haplotype estimation procedures may produce switch errors, which are loci where consecutive heterozygous SNPs are phased incorrectly. These errors may have a dramatic effect on downstream analyses. In order to characterize the sensitiveness of Haplous to such switch errors, we compared haplotypes from individuals in the HapMap database [18] whose haplotype phase was deterministically estimated by using pedigree (phase-known) data to a setting in which the haplotype phase was estimated using a population based method (phase-predicted). The phase-predicted dataset has switch errors from population-based phase estimation, whereas the phase-known dataset can be considered as a dataset without phasing errors.

**Figure 5:**
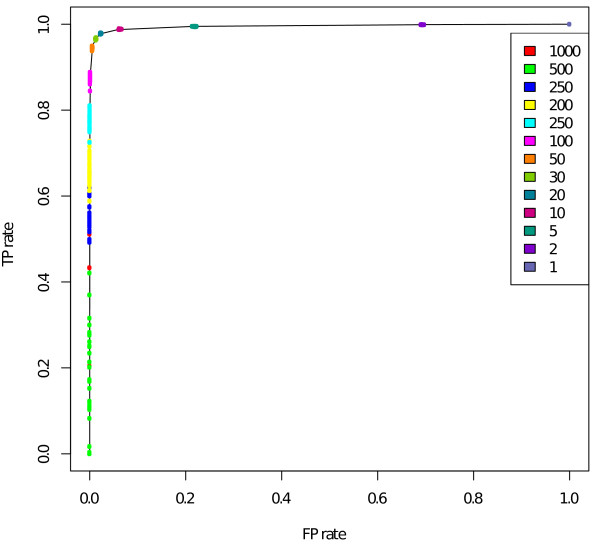
**The ratio of true positive and false positive rates in the HapMap data which include phasing errors**. This graph shows the trade-off between including correct shared haplotypes (SHs) and mismatching markers in the results. The true positive (TP) rate was quantified by the number of markers that were included in SHs similarly in phase-known and phase-predicted datasets, and the false positive (FP) rate was calculated from the mismatching markers that were included in the SHs. We varied the window size *w *from 1 to 2,000, *m *was set to 1.

We compared SHs between non-related individuals in phase-known and phase-predicted datasets. This comparison gives an estimate of the trade-off between finding correct SHs and including false markers in the SHs. Figure 5 shows the ratio of estimated TP and FP rates. The TP rate was quantified by comparing the number of markers where the SHs were captured similarly in the phase-known and phase-predicted datasets. The FP rate is the number of SNPs falsely included in SHs compared to all SNPs that differ between two samples. The same SHs in both datasets were interpreted as TPs, and the SHs only in the phase-known dataset were considered as false negatives (FNs).

We executed Haplous by varying the window size *w *from 2,000 to 1 and allowing one mismatch in the window. Window size *w *= 2,000 did not find any SHs and with *w *= 1 Haplous included all the markers in SHs. Figure 5 shows the ratio of TP and FP rates. When the window size was between 100 and 30, the TP rate was high and the FP rate low. When a very large window size (*w *> 200) was used, SHs seemed to correspond to the real SHs quite poorly, which is shown as a rapid decline in the TP rate. Also, when the window size was smaller (*w *< 30), which means higher tolerance for mismatches, Haplous considers almost all markers as SHs.

### Mutated haplotype in the simulated data

**Figure 6:**
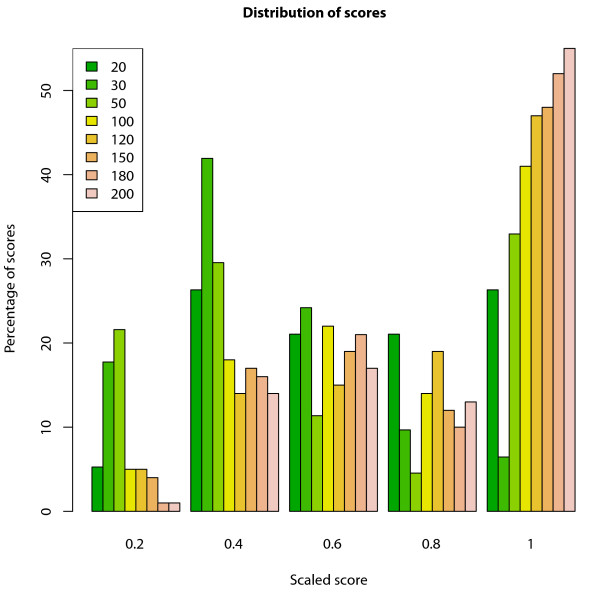
**The distribution of the scores of mutated haplotypes in the simulated data**. A simulated dataset of 11 cases and 250 controls was analyzed using a 100 marker window and requiring 6 cases and 3 controls to share the SH. Scores are scaled between 0 and 1. The mutated shared haplotypes (SHs) from the largest windows received mainly the highest scores, while the window of size 20 received evenly low and high scores.

We applied Haplous to 100 simulated datasets each having 11 cases from an extended pedigree and 250 healthy non-related controls. We extracted different sizes of identical SHs from each of the simulated datasets. The SHs that were left in the dataset after filtering were considered interesting. These interesting SHs were found from each of the simulations, and the mutation was included in most of these results (rows in bold in Table 3). The largest window sizes performed the best; they identified the mutation in every run and the mutated haplotype was the top hit in the SHs of highest frequency. The performance declined with smaller window sizes. The length of interesting SHs increased with the window size. Figure 6 shows the distribution of the scaled scores found with different window sizes. The longest windows created the highest scoring SHs, and the mutation SHs found with a window size of 20 received evenly low and high scores. The longest windows probably find the most specific haplotypes, which also can be seen in the scores.

**Table 3:** Summary of the results from the simulated dataset using different thresholds

		Window sizes							
	
	Threshold	20	30	50	100	120	150	180	200
Percentage of results that included the	3	36	80	96	100	100	100	100	100
mutation (%)	4	23	68	91	100	100	100	100	100
	5	20	62	88	100	100	100	100	100
	**6**	**19**	**62**	**88**	**100**	**100**	**100**	**100**	**100**
	7	19	62	88	100	100	100	100	100
	8	19	62	88	100	100	100	100	100
	9	19	62	88	100	100	100	100	100
	10	19	62	88	100	100	100	100	99
	11	18	62	87	99	99	99	98	99
Percentage of mutation loci in the top hit	3	8	3	18	29	30	37	39	42
(%)	4	13	3	19	28	30	35	37	38
	5	15	3	19	29	31	34	38	41
	**6**	**21**	**3**	**19**	**31**	**35**	**39**	**43**	**45**
	7	21	3	23	33	39	43	48	51
	8	21	5	27	37	43	45	50	54
	9	26	11	31	45	49	54	60	64
	10	26	13	39	55	58	65	68	70
	11	33	26	52	74	80	85	87	87
Mean length of haplotypes (number of	3	18	49	166	470	603	788	849	912
SNPs)	4	18	39	138	375	485	581	634	676
	5	18	37	129	350	416	479	532	572
	**6**	**19**	**37**	**129**	**344**	**408**	**467**	**520**	**559**
	7	19	37	128	344	408	467	520	559
	8	19	37	128	345	411	470	524	561
	9	19	37	128	344	405	458	508	544
	10	19	37	128	344	403	455	500	538
	11	20	37	125	334	383	430	468	491

The threshold for the number of cases was varied in all window sizes. The results where six cases were required to share the haplotype (rows in bold), which corresponds to the lymphoma study, were compared with the BEAGLE results. The percentages of datasets that included the mutation and the percentage of mutated haplotypes being the top hit were calculated from the 100 simulated datasets. The mean length of haplotypes was also calculated from the 100 simulated datasets.

For comparison, we used BEAGLE [14] to identify IBD regions from the data set. The mean length of all significant BEAGLE IBD regions (score < 10^-13^) was 282 markers, and the mutation was included in 9% of the significant IBD regions. Next we took the 50 highest scoring IBD regions from each of the result sets to examine the most interesting IBD regions, resulting in 5,000 best IBD regions. The mean length of these IBD pairs was 1,303 markers. The mutation was found in 46% of the best IBD regions. The mean length of these regions was 1,618 markers. This shows that BEAGLE detected many very long IBD regions between the samples. Long regions, however, are not well suited for localizing the mutations.

### Robustness of Haplous

In order to demonstrate the performance of Haplous in situations where the assumptions in terms of parameter choices or sample groupings are incorrect, we designed three cases that show: 1) the effect of loose and stringent assumptions; 2) the effect of insufficient numbers of controls; and 3) the effect of mixing controls to cases, which corresponds to unrelated phenocopies that are falsely assumed to be distantly related cases.

We studied the effect of loose and stringent parameter values by running the simulation with varying thresholds for cases from 3 to 11. Sensitivity is reduced with increased stringency and the FN rate, as expected (Table 3). With the increased FN rate, we see also increased specificity, since up to 87% of the top hits carried the mutation. Also, the mean length of the haplotypes decreased, which localizes the mutation with higher precision.

In order to evaluate the effect of insufficient numbers of controls, we used 11 cases and reduced the number of controls from 210 to 10 with an interval of 50 controls at a time. We performed the analysis using a 100 marker window and required 6 cases and 3 controls to share the SH. Table 1 shows that the mutated haplotype was identified from all the simulations, and it received the highest ranking more often with low numbers of controls. However, the number of controls had a large effect on precision, which improved with a higher number of controls. This can be seen by the increased length of SHs with the smaller number of controls.

In order to study how often we would get similar results by random, we mixed controls and cases. We used 11 case datasets, each having 11 samples, of which 1 to 10 samples were controls. Corresponding controls were removed from the control data set. We did the analysis using a 100 marker window and required 6 cases and 3 controls to share the SH. Table 2 shows that Haplous does not find FP haplotypes if the data are far from the assumptions in the research setting. In this case the user can clearly see that the assumptions were false since Haplous, instead of FP results, did not return anything. When there were six controls and five cases, Haplous discovered SHs only from 12% of the datasets. Only one of these datasets (8%) included the mutation, which was, however, the top hit in that simulation.

### Lymphoma case study

We applied Haplous analysis to three Finnish families with a possible lymphoma predisposing gene. HL is a B-cell-derived hematopoietic neoplasm that is classified into cHL and NLPHL. Approximately, cHL accounts for 95% and NLPHL for 5% of new cases [35]. Based on recent gene expression studies on tumor cells in both HL subtypes, it seems that cHL and NLPHL are separate, but closely related, disease entities [34]. In addition, the expression pattern of the neoplastic cells in NLPHL is close to that of the cancer cells in TCRBCL [34], which is a subtype of NHL.

We have previously reported a family where four cousins (family 1 in Figure 3) have had the rare subtype of HL, NLPHL, in their early twenties [36]. In the current study, we identified a new NLPHL family with three affected siblings from northern Finland (family 2 in Figure 3) by using a systematic search for related NLPHL patients from the Finnish Cancer Registry and genealogy studies. Interestingly, we were able to connect this family to another family with seven individuals with lymphomas, including two with TCRBCL (family 3 in Figure 3). Both families originated from the same geographical region. The common ancestor of these families was born in the 1670s. Since NLPHL is very rare, and NLPHL and TCRBCL are closely related lymphoma subtypes, it is possible that common genetic factors contribute to lymphoma susceptibility in these three families.

Conventional linkage analysis was not applicable (data not shown) in the combined pedigree of the two families (families 2 and 3 in Figure 3) due to the complex and looped structure and large size of the pedigree together with low DNA sample rate on the affected individuals. Therefore, to detect the putative IBD haplotypes in lymphoma patients from these families we used Haplous. We compared long SHs in lymphoma patients to SHs in unaffected controls to separate the IBD haplotypes from IBS haplotypes and to exclude haploblocks with high linkage disequilibrium (LD) in order to identify interesting regions for further studies.

### Shared chromosomal regions in lymphoma families

We assumed the genetic defect behind lymphoma predisposition is the same in at least two of the families. Thus, considering the number of available samples, we decided to look for overlapping SHs that are present in at least five lymphoma patients, but are not frequently found in unaffected controls. These criteria enabled us to exclude haplotypes that are shared by patients of either family 1 or family 2 only, but allowed the possibility that some patients are phenocopies and do not necessarily share a haplotype with others. To avoid IBS haplotypes, we allowed SHs to exist only in three controls as heterozygous, and did not allow any SHs as homozygous in controls. The pedigrees do not clearly represent a certain mode of inheritance; therefore, six independent analyses were performed where the minimum number of required hetSHs and homSHs in cases was varied to cover all possible combinations of hetSHs and homSHs. A SH with a length of 30 or more SNPs was found in 1,288 different regions. Each SH was given a score based on the number of markers in the SH and on the number of times the cases share a SH. The highest scoring regions found in each analysis using different parameters are shown in Additional file 1.

The SH with the highest score in Additional file 1 was found in the analysis where at least five affected individuals were required to have a hetSH. This region is in chromosome 18, spanning approximately 3 Mbs, and includes 15 genes. The individuals sharing the haplotype are from families 2 and 3. The largest region with the highest number of genes, encompassing 5.6 Mbs and including 137 genes, was found in chromosome 15 using the same parameters. The haplotype in this region is shared by five individuals from family 3. An interesting and high scoring region was also found in chromosome 12, where five individuals have a hetSH and three a homSH. This region is 1.8 Mbs long and contains 37 genes. This region was one of the ten highest scoring regions in three separate analyses, as shown in Additional file 1.

The highest scoring regions in Additional file 1 contain 684 genes, from which we focused on protein-coding genes. Out of the set of 684 genes, 273 had a UniProt code and were considered as protein coding. We analyzed these genes using the SNPs3D [22] text mining method to search for lymphoma-related features. This analysis highlighted seven potentially interesting genes as shown in Additional file 1. *BCL10 *(B-cell CLL/lymphoma 10) is involved in a translocation found in B-cell lymphomas of mucosa-associated lymphoid tissue (MALT lymphomas) [37]. *BCL10 *has also been reported to activate *NF-kB *[37]. *MBP *(myelin basic protein) is expressed in all hematopoietic cells, including B cells [38]. *SIAH1 *(seven in absentia homolog 1) is known to be down-regulated in diffuse large B-cell lymphoma as the result of Epstein-Barr virus infection [39]. *ST8SIA1 *(ST8 alpha-N-acetyl-neuramide alpha-2,8- sialyltransferase 1) contributes to the formation of GD3 ganglioside [40], which is a cell surface molecule involved in various functions of the cell, such as apoptosis, cell growth and adhesion. It is expressed in various tissues, including activated germinal center B cells [41]. *SNRPN *(small nucleolar polypeptide) is an imprinted gene that is methylated in leukemias [42]. *CACNA1H *(calcium channel, voltage-dependent, T type, alpha 1H subunit) encodes a voltage-dependent calcium channel and is located in a region that appears to be hypomethylated in T-cell leukemias [43]. *SPSB3 *(splA/ryanodine receptor domain and SOCS box containing 3) encodes a protein that belongs to a family of socs proteins that contain a SPRY domain, but its gene function is so far unknown.

### Shared haplotypes in the lymphoma dataset

Using results from previous research where significance of SHs has been studied [44], the probability of all cases sharing at least one IBD haplotype, regardless of the disease status, is:

(1) $$1-e^{-\left(d\lambda +k\right)2^{a-d}}\leq \left(d\lambda +k\right)2^{a-d}$$

where *d *is the number of meioses between cases and their common ancestor, *a *is the number of common ancestors (1 or 2), *k *is the number of chromosomes, and *λ *is the expected number of recombinations over these chromosomes. For human data, *k *= 22 and *λ *≈ 35 [45]. Below we also assume that *a *= 2.

Next we generalize Equation 1 to access significance of the high scoring regions of the lymphoma data. The probability that *c *cases out of *n *shared an IBD haplotype, regardless of the disease status, is $\leq \left(\begin{array}{c} n\\ c \end{array}\right)\left(d_{c}\lambda +k\right)2^{a-d_{c}}$, where at least *dc *meioses separates any subset of *c *cases from their common ancestor. The significance that at least *C *cases shares some IBD haplotype is:

$$p\leq \sum_{c=C}^{n}\;\left(\begin{array}{c} n\\ c \end{array}\right)\left(d_{c}\lambda +k\right)2^{a-d_{c}}$$

As *d_c _*<*d_c _*+ 1 we can write:

(2) $$p\leq \left(d_{C}+k\right)2^{a-d_{C}}\sum_{c=C}^{n}\left(\begin{array}{c} n\\ c \end{array}\right)\left(c-C+1\right)2^{C-c}$$

The interesting haplotypes were shared by five to eight samples (Additional file 1). Using the right-hand side of Equation 2, we discovered that finding five IBD SHs is significant at the 5% level if *d_5 _*≥ 27.

Similarly, finding eight SHs is significant if *d_8 _*≥ 24.

If the common ancestor is, on average, at least *x *generations away from all subsets of *C *cases, then *d_C _*must be at least 2*x *+ *C *- 2. This is because there must be two cases that are connected by at least 2*x *meioses, and the remaining cases must each contribute at least 1 to *d_C _*. This simple reasoning shows that when *d_5 _*≥ 27 for five significantly shared haplotypes, the common ancestor is, on average, at least 12 generations away from the samples. For eight SHs the common ancestor must then be, on average, at least 9 generations away. In the lymphoma data, both of these significant (within 95% confidence interval) distances from the common ancestor seem probable by inspecting the partial pedigree of the lymphoma families (Figure 3).

Following the original analysis [44], the length of an IBD region (in Morgans) right and left from the disease loci is exponentially distributed with parameter *d*, if the cases (or their subset) are connected by *d *meioses. So the total length of the IBD region is the sum of these two exponentially distributed random variables. Thus, the expected length of such a region is $\frac{2}{d}$. When *d *increases, the IBD region becomes narrower, but at the same time becomes more significant as well. All of the analysis above has concerned IBD. However, IBS values can only be obtained from the lymphoma data. The separate control data allow filtering out of some IBS regions that are not IBD. Moreover, if the IBS region found is long enough, then it is likely to also be IBD. In light of the analysis above, this depends on the number of meioses *d *connecting the cases. When *d *is sufficiently large, but not too large, the above analysis works also with IBS values.

**Figure 7:**
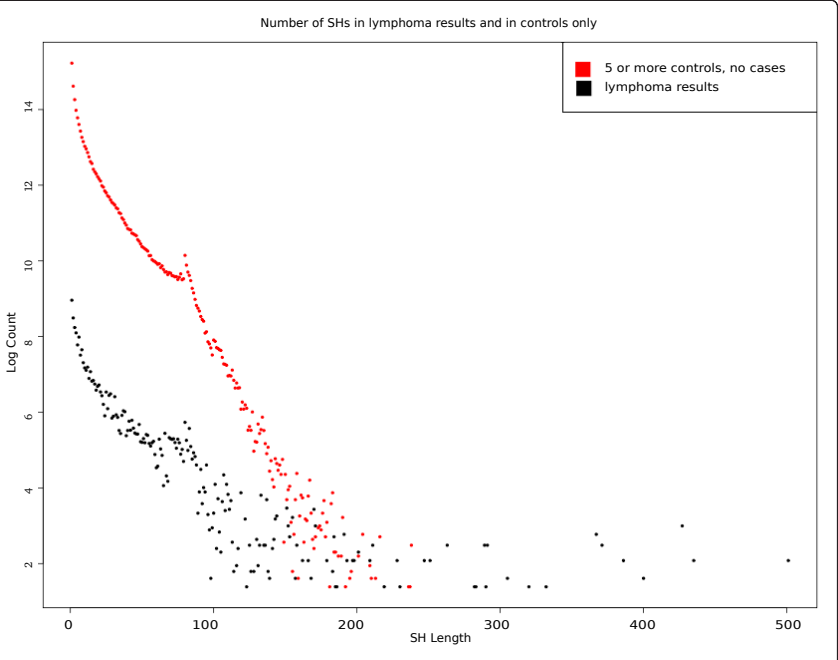
**Distribution of shared haplotype lengths and numbers of shared haplotypes**. The figure shows the distribution of shared haplotypes (SHs) in at least five controls (heterozygous shared haplotype (hetSH) or homozygous shared haplotype (homSH)) in red, and the distribution of the SHs in the lymphoma dataset. SHs from the lymphoma dataset are longer than those in the five controls. These SHs are also rare, as expected.

To evaluate haplotype differences between the lymphoma families and unaffected controls, we compared the length of SHs in the whole dataset, in five or more controls, and only in interesting SHs (five or more cases). Figure 7 shows the number of SHs on a logarithmic scale and their lengths from the six independent analyses (black) compared with the SHs that are shared five times only in controls (red). Since we had 250 controls and only 13 cases, SHs in controls are shared in much higher numbers than SHs in cases. Nevertheless, the length of SHs declines more rapidly in controls than in cases. This was expected, since controls should not have close ancestry and their SHs are from shorter IBS haplotypes.

**Figure 8:**
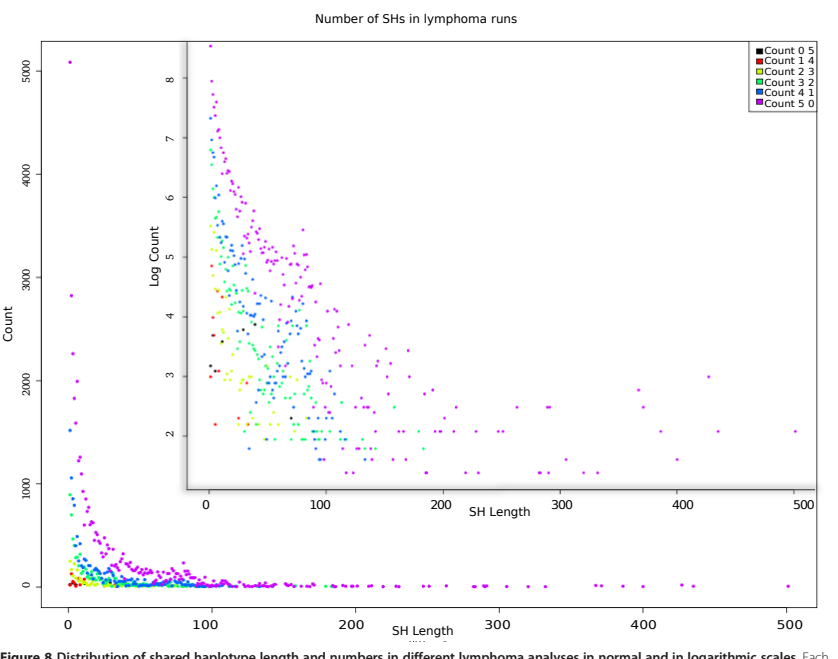
**Distribution of shared haplotype length and numbers in different lymphoma analyses in normal and in logarithmic scales**. Each lymphoma analysis has a different distribution, which is proportional to the number of times the shared haplotype (SH) is shared in the data.

Despite long SHs in cases, we did not pick the most interesting SHs based solely on the length, but performed six analyses on cases to cover all combinations of homozygous and heterozygous haplotypes, and discovered five affected individuals with the same SH. Figure 8 shows the distribution of SH length and number from these analyses, giving the count in normal and logarithmic scales. The distributions of all of the analyses have tails of rarely occurring long SHs, but the tails are in different positions in the x-axis. This is because homozygous samples actually share the haplotype twice and the length of SHs decreases when the number of SHs in the dataset increases. For example, the logarithmic scale shows that SHs shared by five heterozygous samples have the longest SHs, but are also shared the lowest number of times. Furthermore, SHs shared by five homozygous samples (black) are found only in short regions.

## Discussion

We have developed Haplous for haplotype-based analysis and comparison. Haplous enables the search of IBD haplotypes in a group of affected individuals determined by the user in a rule-based manner. The rules and parameters can be adjusted flexibly. Our approach is applicable to studies aiming to identify rare or moderate penetrance genetic determinants that cannot be found by traditional linkage and GWA analyses.

The TP and FP ratio of the SHs identified from the HapMap data indicates that even though accuracy of haplotype estimation has raised some concerns [13], Haplous is able to compensate for possible incorrect switches from the haplotype phasing (Figure 5). In the HapMap test, Haplous achieved a high TP rate in a very limited number of samples. The TP rate declines especially with long window sizes, the most likely cause of which is that HaploRec, similar to many other haplotype phase estimation software, tends to favor common haplotypes in the dataset, which cannot be adapted for. Therefore, haplotype phase should be estimated for cases and controls separately to ensure that haplotypes in the controls do not hide the typical haplotypes in the cases. Haplous uses the number of markers to define the SH length, which is an adaptation to the LD structure in the population, since SNPs in many genotyping arrays are designed accordingly. If the marker density does not take into account the LD structure in the study population, we suggest cleaning the data from markers in LD blocks before running Haplous. If matched population controls are included in the analysis, they usually represent the same LD structure, and the possible bias can be corrected with proper filtering.

Haplous identified the mutated region from the simulated data with high frequency. The simulation followed the setting in the lymphoma study, which shows that our approach is applicable to real-life genome-wide genetic data. The longest windows created the highest scoring SHs, probably because long SHs are more specific for the cases. On the other hand, a small window of 20 markers performed the worst, probably because it finds frequent SHs in both cases and controls. In general, a SH of 20 markers cannot be considered to harbor an IBD region most of the time.

Haplous identified the mutation more efficiently than BEAGLE when we applied the same assumptions that were used in the lymphoma study. BEAGLE's sensitivity in all IBD regions was much lower than the regions returned by Haplous (Table 3); the 50 highest scoring IBD regions of BEAGLE were comparable with the performance of Haplous when using 20 to 30 marker windows. In addition, Haplous had better precision in terms of haplotype length (Table 3). Furthermore, BEAGLE is designed for discovering pair-wise IBD regions, and in the extended pedigree they are usually very long. Therefore, the BEAGLE analysis would require the tedious work of combining multiple IBD regions.

We have shown how the results are affected if the initial assumptions used for setting the parameters are wrong. Table 3 shows how sensitivity and specificity are affected by the parameter stringency. Too stringent values will not capture the whole signal and too loose will include FP regions in the results. The FP results should not be considered as a problem as the findings can be verified using other means, such as next generation sequencing, but a decrease in sensitivity is definitely a problem. Table 1 shows that the matched controls will improve the identification of the mutation. Haplous will find the mutation if the assumptions are close to the 'true values', as they were in this test, but its performance suffers if the number of controls is insufficient. Table 2 shows that Haplous does not find FP haplotypes if the data do not correspond to the research setting. Accordingly, the user can see from the results that the assumptions were false since Haplous, instead of giving FP results, did not return anything. In the borderline case where the data are almost as assumed (five affected cases and six non-related controls in Table 2), it may be difficult to distinguish whether analysis is successful. In general, Haplous performs well even in suboptimal situations. However, it is best to vary the parameters in multiple runs to explore the limitations in the data.

The aggregation of HL in some families has been long acknowledged, but the causes of this clustering are largely unknown. NLPHL is a rare subtype of HL and the presence of several affected family members strongly suggests a genetic factor contributing to disease predisposition. Such families, as we have reported here, provide an excellent opportunity for genetic studies. However, sporadic lymphomas are common and phenocopies further complicate gene identification studies. Therefore, for the reported families, we used Haplous to search for haplotypes that are shared by most patients, but are rare or absent in the control population.

Six different Haplous analyses were run because the pedigrees do not clearly represent a certain mode of inheritance. These analyses covered all combinations of homozygous and heterozygous haplotypes that could lead to five affected individuals having the same SH. This way the smaller families, families 1 and 2, could not produce a hit alone without support from the other families. As the pedigree of family 3 is complex and contains several lymphoma patients, such regions that are shared by five patients from family 3 would also be of interest. As the genome-wide search produced over 1,000 regions with a SH, we decided to focus on the 10 highest scoring regions from each analysis. These regions still included 273 known protein coding genes. In order to further prioritize the gene set, we performed a SNPs3D search to find the genes with lymphoma-related features.

Even though the number of generations from last common ancestor is unknown in the lymphoma dataset, we have estimated that SHs shared by five cases are true IBD haplotypes with 95% confidence. Figures 7 and 8 show that, as expected, we found longer SHs in lymphoma cases than in the general population. Also, the number of SHs declines rapidly with the SH length. As we showed with the simulated data, the mutated haplotypes in the related samples are longer than those in the population in general. Therefore, we can conclude that long rare SHs in lymphoma cases are not seen in the population and may well carry the disease predisposition mutation.

With literature mining, we found seven genes that are frequently associated with hematopoietic cells and malignancies, lymphomagenesis or lymphoma-related pathways. The most interesting of these seven genes is *BCL10*, which is known to be disrupted in other kinds of lymphomas [37]. A link between *SIAH1 *and NHLs was also found in the literature, when Epstein-Barr virus infection had been detected in the tumor [39]. Similar direct links to lymphomas could not be found for the rest of these genes. One reason for this is that the principal phenotypes we study here are NLPHL and TCRBCL, which are rare lymphoma subtypes and not much is known about their molecular background. No gene hits were found when 'nodular lymphocyte predominant Hodgkin lymphoma', 'T-cell rich B-cell lymphoma' or 'histiocyte rich B-cell lymphoma' were used as keywords. This could suggest that the genetics of these lymphoma types are still largely unknown. Clearly, the putative gene harboring a predisposing variant can also be a gene that has not yet been implicated in the literature to be related to hematological malignancies or B-cell development, and the SNPs3D search must be considered only as a supporting method to prioritize candidate genes for more detailed analyses.

The distantly related families 2 and 3 had the highest scoring SHs. This could indicate that the genetic defect underlying the cancer predisposition in family 1 is possibly not of the same ancestral origin as in the other families. It is also possible, that the genetic locus shared by all three families is located in a moderately short genetic region, where the score would be lower, or embedded within a haplotype that is relatively common in the population. Due to the uninformativeness caused by the missing DNA of some samples, some of the interesting regions can be false positive hits. In general, Haplous allows more stringent criteria for handling controls and uninformativeness than what we have used in our analysis. In the lymphoma study, however, the haplotypes of some of the key individuals have been predicted using SNP data from their relatives, resulting in a considerably large number of genetic regions where the phase-known haplotype cannot be estimated. Thus, we decided to tolerate FP SHs rather than lose information, especially as the effect of the FPs can be narrowed down in the future by combining the results with other platforms, such as gene expression studies and large scale sequencing efforts. Results from Haplous enable targeted next generation sequencing of putative disease-associated regions, and help focus the analysis of whole genome or exome sequencing data to particular loci; a key feature considering the abundance of variants detected by these methods.

Taken together, Haplous can be used to scrutinize next generation sequencing results that contain plenty of irrelevant information and errors, especially when other SNP array-based methods are not applicable. This is an important feature in settings where the sample sizes are small. Even though the number of FPs increases with small sample numbers, this cannot be seen as a major obstacle as novel high-throughput sequencing technologies are able to cover multiple large regions with relatively low cost. The outcome of Haplous is a ranked set of candidate regions that fulfill the criteria determined by the user. The results are easy to interpret, which increases the reproducibility that has been a challenge in previous haplotype-based tests [4]. The user can also choose whether to emphasize the haplotype length or the number of cases sharing a haplotype when the score for each region is created. Haplous is flexible and allows various testing of various inheritance models and datasets such as a recessive family-based inheritance model, IBD analyses in multiple pedigrees of any size, homogenous population samples, or any combination of these.

## Conclusions

We have developed a novel computational method, Haplous, which is a rule-based haplotype comparison method for flexible analysis of haplotypes with high accuracy in groups of individuals, enabling these haplotypes to be further used to locate disease-causing mutations. We used Haplous to identify haplotypes that are common to patients in three lymphoma families to which linkage analysis could not be applied. Several interesting loci were identified, and these results can be integrated with the patient data produced by other high-throughput approaches, such as gene expression and sequence information.

## Abbreviations

cHL: classical Hodgkin lymphoma; FN: false negative; FP: false positive; GWA: genome-wide association; hetSH: heterozygous SH; HL: Hodgkin lymphoma; homSH: homozygous SH; IBD: identical by descent; IBS: identical by state; LD: linkage disequilibrium; NHL: non-Hodgkin lymphoma; NLPHL: nodular lymphocyte predominant Hodgkin lymphoma; SH: shared haplotype; SNP: single nucleotide polymorphism; TCRBCL: T-cell/histiocyte rich B-cell lymphoma; TP: true positive.

## Competing interests

The authors declare that they have no competing interests.

## Authors' contributions

SK developed Haplous, and was responsible of the bioinformatics of the study and wrote the corresponding sections of this article. SS was responsible for the lymphoma study and wrote the corresponding sections of this article. RL was responsible for co-supervising the algorithm development and the data analysis and reviewed and modified the manuscript. PR was responsible for data simulation, evaluating the significance of the lymphoma results and wrote the corresponding sections. PV produced the lymphoma SNP data and family-based haplotypes and reviewed the manuscript. LAA contributed to the lymphoma case study and to the writing of the manuscript. SH initiated and supervised the project, and contributed to the writing of the manuscript. All authors have read and approved the manuscript for publication.

## Supplementary Material

Additional file 1 **The results of all six different Haplous analyses performed on lymphoma families**. If the analysis produced more than 10 hits longer than 30 SNPs, only the 10 highest scoring shared haplotypes (SHs) are shown. The number of heterozygous SHs (hetSHs) and homozygous SHs (homSHs), the region boundaries, and the individuals who share the haplotype are also shown. Ensembl release 59 was used as the reference build. The codes for individuals in families 1 to 3 are the same as in Figure 3. Separately depicted SHs can partially overlap as affected individuals can share different parts of the haplotype within a region. Click here for file
